# MicroRNA-148b Inhibits the Malignant Biological Behavior of Melanoma by Reducing Sirtuin 7 Expression Levels

**DOI:** 10.1155/2020/9568976

**Published:** 2020-11-20

**Authors:** Rui Sun, Meiliang Guo, Xiaojing Fan, Qinqin Meng, Dingfen Yuan, Xinrong Yang, Kexiang Yan, Hui Deng

**Affiliations:** ^1^Department of Dermatology, Shanghai Jiao Tong University Affiliated Sixth People's Hospital, Shanghai 200233, China; ^2^Department of Dermatology and Venereology, Huashan Hospital, Fudan University, Shanghai, China

## Abstract

There is growing evidence that microRNA-148b (miR-148b) can inhibit the growth of malignant cells while sirtuin 7 (SIRT7) may perform its carcinogenic effect by deacetylating H3K18. This study investigated the mechanism of miR-148b/SIRT7 on how it affects the malignant biological behavior of melanoma. It was established that the expression of miR-148b was downregulated in melanoma while that of SIRT7 was upregulated but negatively regulated by miR-148b through binding to the 3′UTR of SIRT7. Ectopic expression of miR-148b reduced the proliferation, migration, and invasion of melanoma cells, but SIRT7 reversed these functions of miR-148b. Moreover, tumor growth and metastasis experiments showed that miR-148b could significantly suppress proliferation and metastasis of melanoma *in vivo*. Overall, miR-148b inhibits the malignant biological behavior of melanoma by reducing the expression level of SIRT7. The development of miR-148b as a novel potential therapeutic approach for melanoma may be possible in the future.

## 1. Introduction

Melanoma is a prevalent malignant tumor. Currently, sensitization on melanoma reiterates the importance of early detection due to the high feasibility of cure rates after surgical excision [1, 2]. Early detection also aids in the prevention of metastatic disease and death [3, 4]. However, despite promising progress in surgical resection, radiotherapy, and chemotherapy of melanoma, long-term survival remains extremely low due to recurrence and metastasis [5].

MicroRNAs (miRNAs) are small endogenous, noncoding RNA molecules that can degrade or inhibit target mRNAs at the posttranscriptional level [6–8]. A single miRNA can target hundreds of mRNAs and result in the silencing of many target genes. miRNAs often act as tumor suppressors or oncogenes and have been identified as therapeutic targets for cancer treatment [9]. miRNA-148b is involved in the deterioration of different types of tumors and has been shown to act as a cancer-suppressing factor in hepatocellular carcinoma, cervical cancer, and gastric cancer [10–14].

Sirtuins are proteins that have various enzymatic activities, including NAD^+^-dependent deacetylation and regulation of genome stability, metabolism, and lifespan [15–17]. There are 7 subtypes of sirtuins (SIRT1-7). SIRT7 is mainly located in the nucleoli and selectively binds to target genes at their promoter regions and controls RNA polymerase I transcription [18]. In prostate carcinomas and gastric cancer, SIRT7 overexpression is associated with cancer phenotypes and metastatic diseases [19–21]. SIRT7 causes carcinogenic effects by deacetylating H3K18; this weakens the transcription of target genes linked to tumor suppression. Additionally, SIRT7 upregulates rRNA synthesis, to meet the increased demand for ribosomes in rapidly growing tumor cells [22, 23]. However, the role of SIRT7 in melanoma remains poorly elucidated.

Through various studies done, there has been cumulative evidence indicating that many miRNAs inhibit the growth and invasion of a malignant cell by controlling adiposity and apoptosis through targeting SIRT7 [21, 24–26]. A recent study shows that miR-148b contributes to the regulation of cardiomyocyte apoptosis by targeting SIRT7 [27]. These studies inspired the need to explore the roles of miR-148b and SIRT7 in melanoma and the potential relationship between them.

In this study, we demonstrated that miR-148b acts as an antioncogene in melanoma by reducing the expression level of SIRT7. SIRT7 was highly expressed in melanoma tissues than in the control group. The deficiency of miR-148b resulted in the migration, invasion, and proliferation of melanoma cells. In conclusion, miR-148b inhibits the malignant biological behavior of melanoma by reducing the expression level of SIRT7.

## 2. Materials and Methods

### 2.1. Recruitment of Patients for the Study and Specimen Collection

Human samples were collected from 15 patients diagnosed as melanoma and 14 patients diagnosed as benign nevi according to pathological findings at Shanghai Jiao Tong University Affiliated Sixth People's Hospital. This study was approved by the Ethics Committee of Shanghai Jiao Tong University Affiliated Sixth People's Hospital. Each patient signed an informed consent form before participating in the experiment.

### 2.2. Cell Culture

A375, MEWO, and MV3 cell lines were purchased from the Institute of Biochemistry and Cell Biology, The Chinese Academy of Sciences (Shanghai, China). A375 and MV3 cells and human epidermal melanocytes were cultured in Dulbecco's modified Eagle's medium (DMEM) containing 10% fetal bovine serum (FBS), while MEWO cells were cultured in minimum essential medium (MEM) containing 10% FBS at 37°C and 5% CO_2_ in an incubator.

### 2.3. RNA Extraction and Real-Time Quantitative PCR (RT-qPCR)

Total RNA was isolated from the melanoma tissue, benign nevus tissue, melanoma cell lines, and normal human epidermal melanocytes using TRIzol reagent (TaKaRa, Dalian, China). Complementary DNA (cDNA) was synthesized using a Mir-X MicroRNA First-Strand Synthesis Kit (TaKaRa). The ratio of SIRT7 and miR-148b mRNA expression was calculated by 2^-*ΔΔ*CT^ (ΔCT = targetgeneCT − internalreferencegeneCT). The cDNA was then diluted for RT-qPCR analysis using SYBR Green Fast qPCR Mix (RR430A) with specific primers. The expression of miR-148b in cells was determined using U6 as an internal reference according to the relative quantitative method. The sequences are listed in Table 1. The housekeeping gene GAPDH was used as an internal control to detect SIRT7 expression. Reactions were conducted by an Applied Biosystems 7500 Fast Real-Time PCR System. Primer sequences are listed in Table 2.

### 2.4. MicroRNA Transfection of A375 Cells

The miR-148b mimics and negative control (NC) mimics and miR-148b inhibitor and NC inhibitor were synthesized by GenePharma (Shanghai, China). A375 cells were inoculated into 6-well plates, and after 24 hours, the cells were transfected with the miR-148b mimic or inhibitor using Lipofectamine 2000 (Invitrogen) as per the manufacturer's protocols.

### 2.5. Plasmid Construction

Wild-type and mutant SIRT7 reporter plasmids were constructed with the help of GenePharma (Shanghai, China), and a Promega Dual-Luciferase® Reporter (DLR) Assay System was purchased from Promega (Madison, WI). The sequences are listed in Table 3.

### 2.6. Bioinformatics for Identification of the Expression Level of SIRT7 and miR-148b in Melanoma and Luciferase Reporter Assay

To investigate the expression level of SIRT7 in melanoma, gene counts and expression data of 51 primary melanoma and 27 common acquired nevus tissue samples from GSE98394 were downloaded from the GEO database (https://www.ncbi.nlm.nih.gov/geo). Differentially expressed genes between the two groups were calculated using the edgeR package in R (version 3.6.3), and the top 50 of them were presented in a heat map using the pheatmap package. Expression data of SIRT7 was obtained and subsequently analyzed with GraphPad Prism 7.

TargetScan, miRbase, and NCBI were used for predicting whether or not SIRT7 is the target of miR-148b. A735 cells were transfected with the miR-148b mimic and SIRT7 wild-type and mutant reporter plasmids. After 3 days, fluorescence intensity analysis was performed using the Dual-Luciferase Reporter Assay System (Promega). The fluorescence intensity ratio was calculated as follows: ratio = Fireflyluciferase/Renillaluciferase.

### 2.7. Western Blot Analysis

A BCA Protein Assay Kit (Tiangen, China) was used to measure the concentration of proteins extracted from cell lysis. The protein sample (40 *μ*g) was separated on 10% sodium dodecyl sulfate- (SDS-) polyacrylamide gels and then transferred to a polyvinylidene fluoride (PVDF) membrane. A solution of 5% nonfat milk and Tris-buffered saline-Tween 20 (TBST) was used to block the PVDF membranes. The membranes were then incubated with specific antibodies against SIRT7 (1 : 1000 dilution, PB0376, Boster, China) overnight at 4°C. An anti-goat IgG-HRP secondary antibody (BA1060, Boster) was then applied to the membranes for 1 hour at room temperature. An enhanced chemiluminescence assay (Pierce, Rockford, USA) was used to visualize the immunoblots. Image-Pro Plus software (version 6.0; Media Cybernetics, Bethesda, USA) was used to image and analyze the blots.

### 2.8. Immunofluorescence (IF) Staining

To determine the expression level and position of SIRT7, paraffin-embedded sections were evaluated by IF staining with a rabbit monoclonal anti-SIRT7 antibody. The 4 *μ*m thick tissues were dewaxed and antigen retrieval, followed by endogenous nonspecific antigen blocking. An anti-SIRT7 primary antibody (1 : 100 dilution, PB0376, Boster) was applied at 4°C overnight. On the following day, a donkey anti-rabbit IgG secondary antibody (Alexa Fluor 488; 1 : 400 dilution, ab150073, Abcam, USA) was added to the sections. The NC group was treated with the same procedures described above, but the antibody was replaced by PBS.

### 2.9. Fluorescence In Situ Hybridization (FISH)

Paraffin sections (4 *μ*m) from 6 cases of intradermal nevus and 14 cases of melanoma were selected. The probe sequence was hsa-miR-148b5′-Cy3-ACAAAGUUCUGUGAUGCACUGA-3′. A mixture of hydrogen peroxide and methanol (1 : 9) was added to the liquid, and the slices were soaked in the mixture for ten minutes. The slices were then washed in diethyl pyrocarbonate (DEPC) water 3 times for 3 minutes for each wash and then placed in a wet box and covered with 0.2 mol% hydrochloric acid at room temperature for 15 minutes. Then, the samples were washed with DEPC water twice for 3 minutes for each wash. Protease K was used to cover the tissue in DEPC water twice for 3 minutes for each treatment. The protease K-covered tissue was then fixed in paraformaldehyde for ten minutes. The slices were placed in a wet box, and a hybridization solution was added to cover the tissue for 1 hour. Then, 500 ng/ml probes were applied to the slices, and the slices were then placed in a hybridization oven for 48 hours. The nonspecific probe was removed by washing with 0.5X saline-sodium citrate (SSC) buffer containing 50% formamide at 37°C. DAPI nuclear staining was conducted for 5 minutes.

### 2.10. Stable Infection

Lentiviruses (GV209) encoding miR-148b and SIRT7 (LV-hsa-miR-148b and LV-SIRT7, respectively) were purchased from GenePharma. A375 cells were inoculated in 6-well plates, and after 24 hours, 2 *μ*l of lentivirus was added to each well (multiplicity of infection (MOI) = 10). Puromycin and bleomycin were used to screen stably infected cell lines. After 3 days, the fluorescence intensity reached 80%, indicating a successful infection.

### 2.11. Cell Migration and Invasion Assays

Migration assays were performed in Transwell Permeable Supports (8.0 *μ*m; Costar 3422; Corning, NY, USA). In a 24-well plate, 500 *μ*l of complete medium was added to the lower layer of the chamber. 6 × 10^4^ cells transfected with miR-148b mimic or control were suspended in 500 *μ*l of serum-free medium and added to each pore of the transwell inserts. After 24 hours of incubation, the migrated cells in the lower surfaces of the membranes were fixed with methanol and stained with crystal violet. Cells were observed in a light microscope and counted. The number of cells passing through the chamber after 24 hours indirectly indicated the difference in the migration ability.

To perform the invasion assay in a 24 well plate, 40 *μ*l of diluted Matrigel (BD Biosciences, San Jose, CA, USA) was added to the bottom of the chamber. A total of 500 *μ*l of complete medium was added to the lower layer of the chamber as a chemoattractant. Next, 6 × 10^4^ cells were suspended in a serum-free medium and the plates were incubated for 24 hours. The cells were stained with crystal violet and observed and counted under a light microscope. The number of cells passing through the chamber after 24 hours indirectly indicated the difference in the invasion ability.

### 2.12. Cell Proliferation Assays

Cell proliferation was detected using a cell counting kit-8 assay (Dojindo, Kumamoto, Japan) according to the manufacturer's protocol. After digestion of the transfected cells, 3000 cells per well were inoculated into 96-well plates, with 5 wells for each group. Cell absorbance was measured at 24, 48, 72, and 96 hours after plating. The data collected was plotted as a proliferation curve. The absorbance of each well was measured at a wavelength of 490 nm by an enzyme label detector (Thermo Fisher Scientific, Rockford, IL, USA).

### 2.13. Colony Formation Assays

Transfected melanoma cells were inoculated into 6-well plates, and the cells were evenly distributed by shaking the plates from left to right. The cells were cultured in an incubator for two weeks. After two weeks, the cells were fixed with paraformaldehyde for 15 minutes and stained with crystal violet for 15 minutes. The clones were counted under a microscope, and clones with more than 50 cells were considered to be effective. The colony formation rate was calculated using this equation: colonyformationrate = numberofclones/numberofcellsinoculated × 100%.

### 2.14. Tumor Growth and Metastasis In Vivo

To study the effect of miR-148b on tumor growth and metastasis *in vivo*, nude mice (4 weeks, male) housed at the Animal Experiment Center at The Sixth People's Hospital Affiliated with Shanghai Jiao Tong University (Shanghai, China) were used. A total of 1 × 10^7^ A375 cells were stably transfected with the hsa-miR-148b lentiviral plasmid or hsa-miRNA-148b/SIRT7 lentiviral plasmid or lentiviral vectors (control group). These transfected cells were then injected subcutaneously into nude mice (*n* = 5, one group). Subsequently, all the mice were housed for three weeks under the same conditions. Then, tumor volume and weight were measured and recorded every 5 days and calculated as follows: *V*(mm^3^) = 0.5 × *a* × *b*^2^ (*a* = maximum length of the diameter and *b* = maximum transverse diameter). A375 cells (1 × 10^5^ cells in 50 *μ*l PBS) were injected into the tail vein of mice (*n* = 5, one group). These mice were housed for 6 weeks under the same conditions. At the end of the experiment, the mice were sacrificed, and the lungs were dissected to count the metastasized colonies. All animal experiments were done in compliance with the ARRIVE guidelines and were carried out per the EU Directive 2010/63/EU for animal experiments.

### 2.15. Statistical Analysis

The GraphPad Prism 5.00 (GraphPad Software) statistical software was used to analyze the data. The ratio of SIRT7 and miR-148bmRNA gene expression was calculated by 2^-*ΔΔ*CT^ (ΔΔCT = targetgeneCT − internalreferencegeneCT). Student's *t*-test was performed for analysis of the mean of two independent samples, while variance analysis was used for comparisons of multiple sample means. All statistical analysis was conducted by bilateral testing. *P* < 0.05 indicated that a difference was statistically significant. All experiments were repeated three times.

## 3. Results

### 3.1. The Level of miR-148b Was Downregulated While SIRT7 Expression Was Upregulated in Melanoma

**Figure 1:**
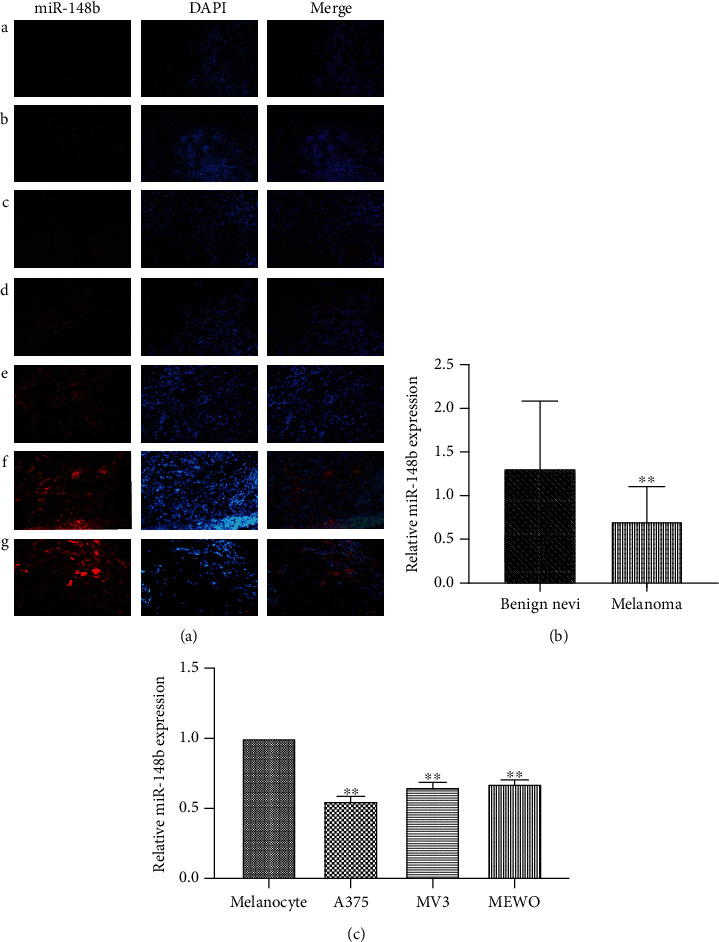
Decreased expression of miR-148b in melanoma. (a) The expression levels of miR-148b in melanoma tissues (A–E) and benign nevi (F and G) measured by FISH, ×400 magnification. (b) The expression level of miR-148b in benign nevus and melanoma tissues measured by RT-qPCR. (c) The expression level of miR-148b in melanocyte, A375, MV3, and MEWO measured by RT-qPCR. ^∗∗^Significant difference at *P* < 0.05.

To explore the potential relevance of miR-148b and SIRT7 in melanoma, gene expression profiles of miR-148b and SIRT7 in melanoma tissues and the control group were first determined by FISH, IF, and RT-qPCR. The fluorescence intensity showing the expression of miR-148b in melanoma tissues was too low to detect (Figure 1(a), A–E) whereas it was higher in the benign nevi (Figure 1(a), F and G). Moreover, the results of RT-qPCR also showed that miR-148b expression was significantly downregulated in melanoma tissues compared with benign nevus tissues (Figure 1(b)). Moreover, we examined the expression of miR-148b in melanoma cell lines and melanocytes. As shown in Figure 1(c), the expression of miR-148b was also markedly decreased in the melanoma cell lines. Together, our studies indicated that miR-148b was significantly downregulated in melanoma.

**Figure 2:**
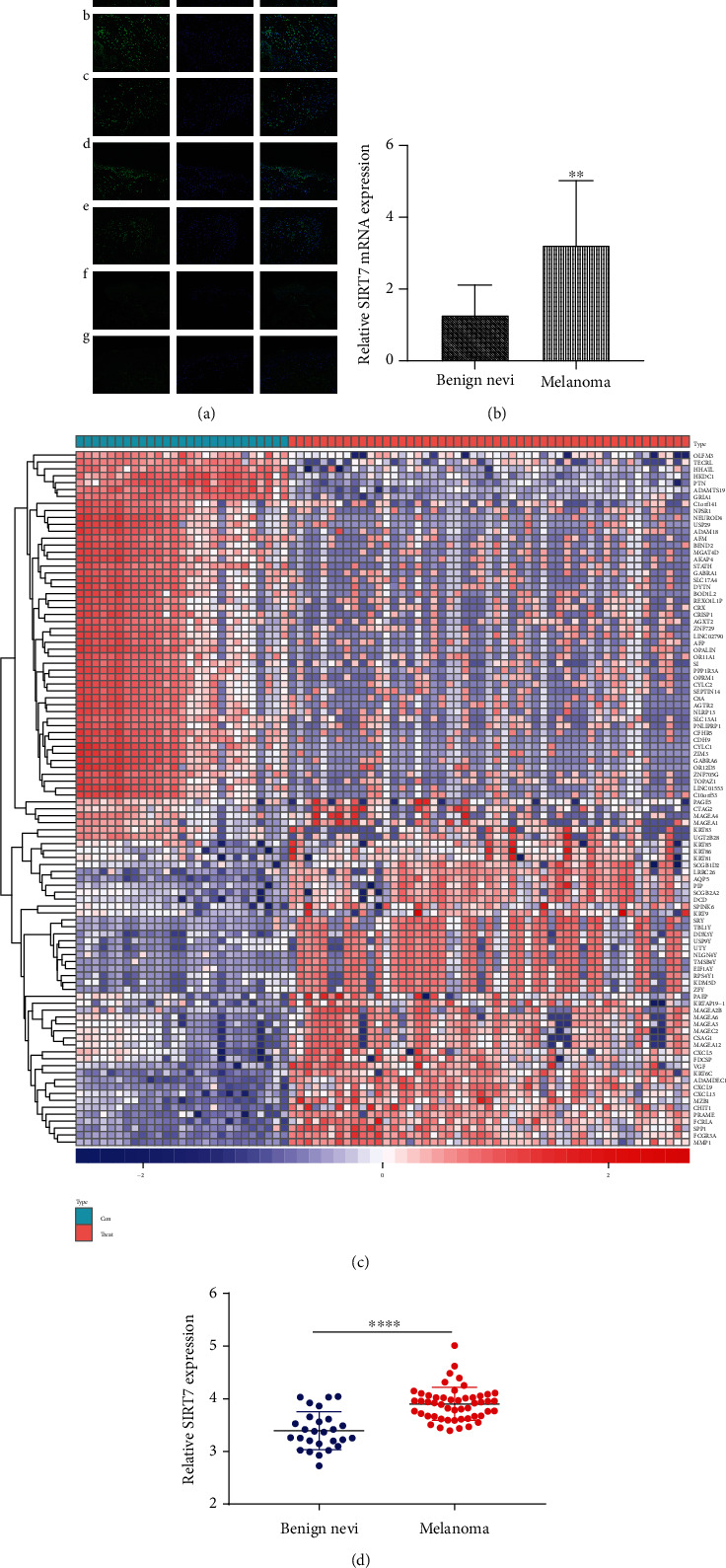
Upregulated expression of SIRT7 in melanoma. (a) The expression levels of SIRT7 in melanoma tissues and benign nevi measured by immunofluorescence staining: A–E: melanoma; F and G: benign nevi; ×400 magnification. (b) The mRNA expression level of SIRT7 in benign nevus and melanoma tissues measured by RT-qPCR. (c) Heat map of top 50 differentially expressed genes in GSE98394 coloring the sample groups, the depth of color represents the expression value of the gene. (d) Relative expression of SIRT7 in normal and tumor group in GSE98394. ^∗∗^Significant difference at *P* < 0.05.

As shown in Figure 2, the fluorescence intensity indicating SIRT7 expression was higher in melanoma tissues (Figure 2(a), A–E) than in the benign nevi (Figure 2(a), F and G), where the intensity was too weak to detect. Also, SIRT7 was primarily located in the nucleus as shown by the IF results. The results of RT-qPCR also indicated that SIRT7 was significantly overexpressed in melanoma tissues compared to benign nevus tissues (Figure 2(b)). The expression heat map of the top 50 differentially expressed genes in the 51 primary melanoma and 27 common acquired nevus tissue samples was as shown (Figure 2(c)), and the relative expression of SIRT7 of the two groups was as shown (Figure 2(d)). These results suggest that the expression of SIRT7 was markedly elevated in melanoma tissues.

### 3.2. SIRT7 Is Negatively Regulated by miR-148b by Binding to the 3′UTR of SIRT7

**Figure 3:**
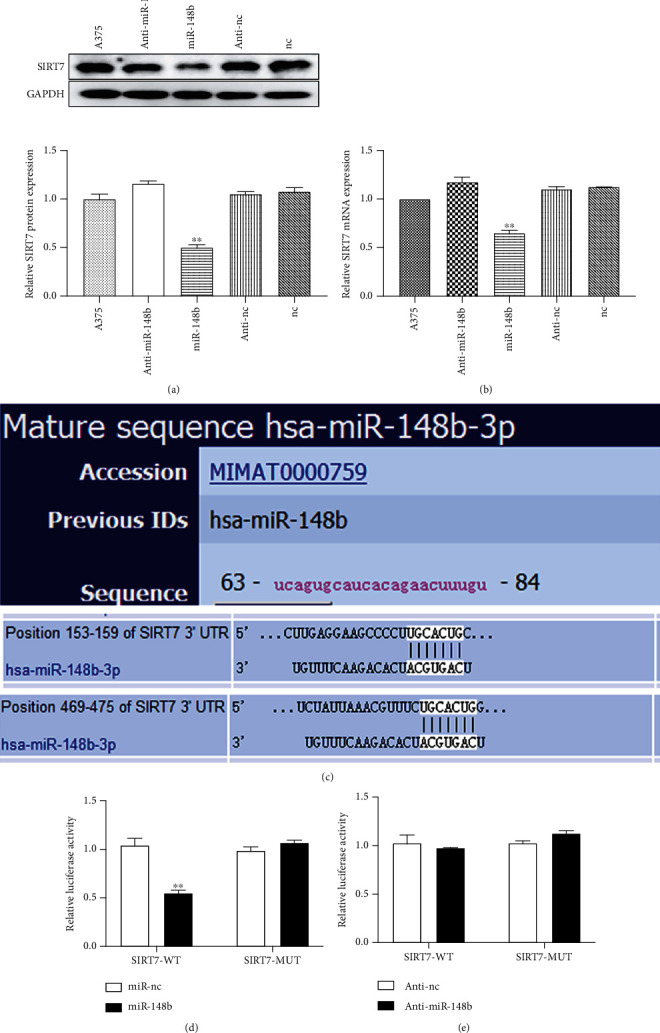
SIRT7 is negatively regulated by miR-148b. A375 cells transfected with miR-148b mimic, miR-148b inhibitor, or empty vector and the level of SIRT7 measured by (a) Western blot and (b) RT-qPCR. TargetScan results of miR-148b (c). The luciferase activity of SIRT7 in the mutant 3′UTR of SIRT7 and WT A375 cell after transfection with (d) miR-148b mimic, (e) miR-148b inhibitor, or empty vector. ^∗∗^Significant difference at *P* < 0.05.

To investigate the potential relationship between miR-148b and SIRT7, we transfected miR-148b mimics or inhibitor in A375 cells. The transfection of miR-148b mimics significantly downregulated the expression of SIRT7. On the contrary, there was no significant difference between the miR-148b inhibitor and control groups (Figures 3(a) and 3(b)). These results indicated that miR-148b inhibits the expression of SIRT7.

Then, we performed bioinformatics analysis to explore the underlying mechanism of the antitumor effect of miR-148b. From the results, it was found that SIRT7 may be the target of miR-148b and that miR-148b has two binding sites within the 3′UTR of SIRT7 (Figure 3(c)). The results of the luciferase reporter assay showed that miR-148b could effectively bind to the wild-type 3′UTR of SIRT7 but not on the mutant 3′UTR of SIRT7. Consequently, there was a significant impact on the luciferase reporter activity (*P* < 0.05, Figures 3(d) and 3(e)). Therefore, miR-148b inhibits expression of SIRT7 by binding to the 3′UTR of SIRT7, which is a direct target of miR-148b.

### 3.3. Ectopic Expression of miR-148b Reduces Migration, Invasion, and Proliferation of Malignant Cells While SIRT7 Reverses the Inhibitory Effects

**Figure 4:**
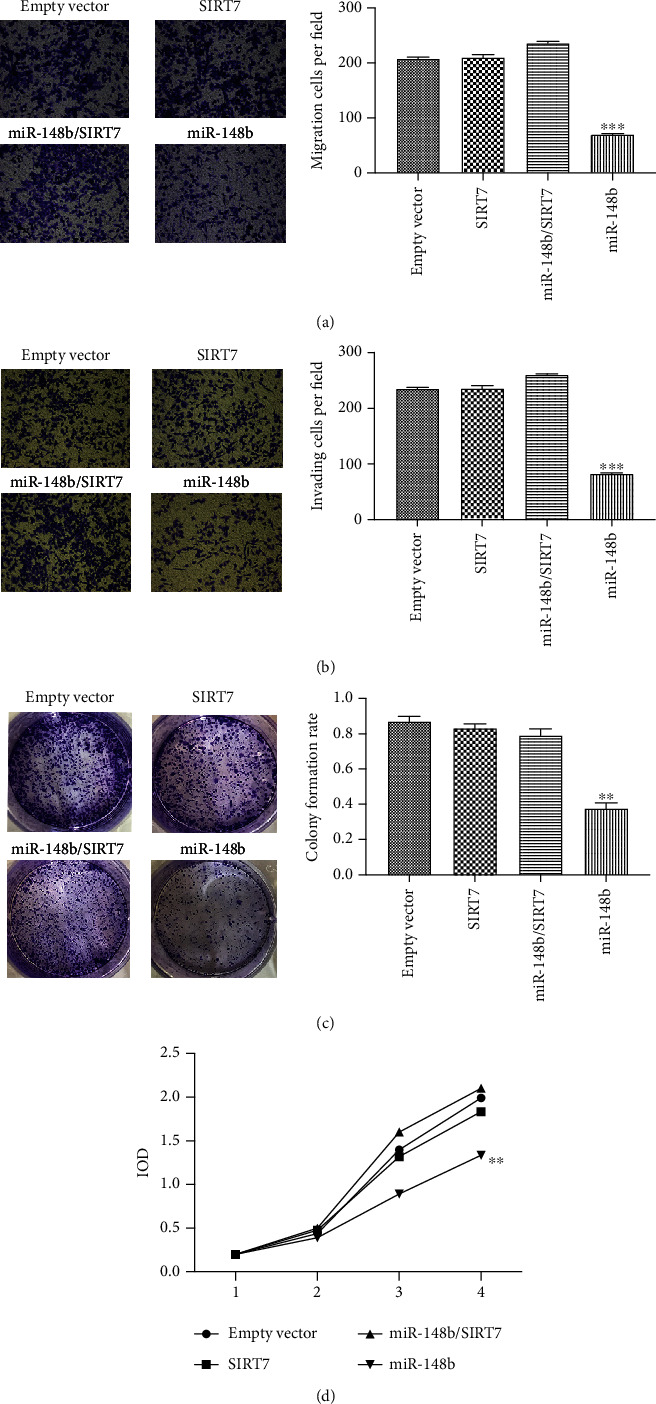
Effect of ectopic expression of miR-148b and SIRT7 on melanoma cells. The (a) transwell migration, (b) Matrigel invasion, (c) colony formation, and (d) cell CCK-8 (proliferation) assay performed in A375 cell stably transduced with LV-hsa-miR-148b or LV-SIRT7 or both LV-hsa-miR-148b and LV-SIRT7, ×200 magnification. ^∗∗^Significant difference at *P* < 0.05 and ^∗∗∗^significant difference at *P* < 0.01.

To investigate the biological functions of miR-148b and SIRT7 in melanoma cells, we performed gain-of-function and loss-of-function experiments in A375 cells stably transduced with LV-hsa-miR-148b; LV-SIRT7 was transfected to A375 cells. The cell migration and invasion assays demonstrated that the number of migrating and invasion cells in the group transfected with LV-hsa-miR-148b was significantly lower than in the other groups (Figures 4(a) and 4(b)). Colony formation assays demonstrated that the rate of colony formation and the number of cell proliferation in the group overexpressing miR-148b were lower than those in the LV-SIRT7 or empty vector-transfected groups (Figures 4(c) and 4(d)). However, transwell assays revealed that groups transfected with both LV-hsa-miR-148b and LV-SIRT7 showed no significant difference in migration, invasion, or proliferation ability. These results confirmed that miR-148b significantly reduced the migration, invasion, and proliferation ability of the melanoma cells but SIRT7 reversed the inhibition effect of miR-148b.

### 3.4. The Effects of miR-148b and SIRT7 on the Proliferation and Metastasis of Melanoma In Vivo

**Figure 5:**
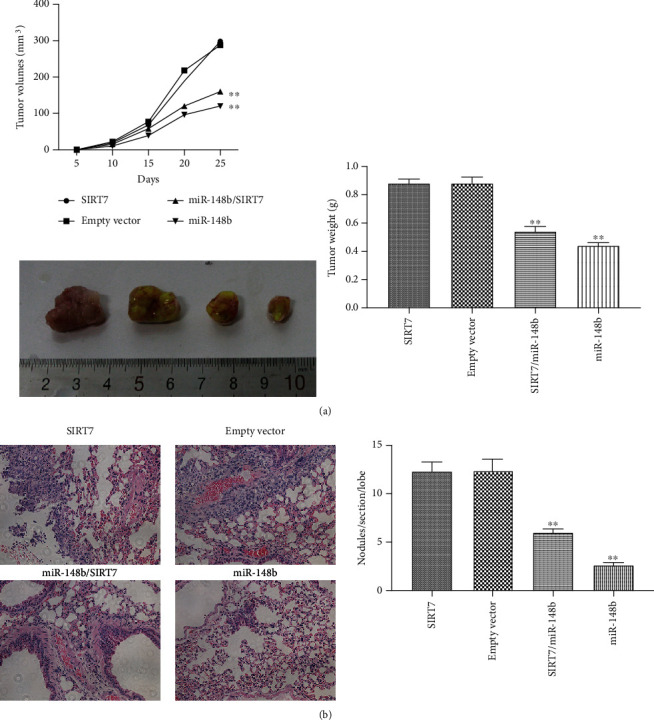
Effects of miR-148b and SIRT7 in melanoma in vivo. (a) Growth curves of tumors after subcutaneous injection of A375 cells stably transduced with empty vector or LV-hsa-miR-148b or LV-SIRT7 or both LV-hsa-miR-148b and LV-SIRT7. (b) The average number of metastases formed per lung section per lobe along with representative hematoxylin and eosin- (H&E-) stained sections and images of lungs metastases, ×400 magnification. ^∗∗^Significant difference at *P* < 0.05.

To further confirm the function of miR-148b *in vivo*, a tumor transplant experiment was performed. Here, A375 cells were stably transfected with the LV-hsa-miR-148b or LV-hsa-miRNA-148b/SIRT7 were injected subcutaneously into nude mice. In the four groups of nude mice transplanted with malignant cells, the two groups overexpressing miR-148b (with or without SIRT7 overexpression) had lower weight and smaller volume of the tumor than the LV-SIRT7 and LV-control groups (Figure 5(a)). Similar to the results reported above on weight and volume of the tumor, lung metastases were significantly lower in the cotransfection group and group transfected with miR-148b (Figure 5(b)). The nude melanoma mice overexpressing miR-148b had the least tumor weight and volume and minimal metastasis.

## 4. Discussion

The present study demonstrated for the first time that miR-148b expression was significantly downregulated while SIRT7 was upregulated in human melanoma cells unlike in melanocytes and benign nevi. The results of the study showed that SIRT7 was negatively regulated by microRNA-148b binding on the 3′UTR of SIRT7. Ectopic expression of miR-148b reduced the proliferation, migration, and invasion of cells while SIRT7 reversed these inhibitory effects *in vitro*. Moreover, *in vivo* experiments demonstrated that miR-148b significantly inhibited the volume, weight, and lung metastases of subcutaneous tumors. The findings of these experiments suggested that miR-148b acts as a suppressor of the malignant biological behavior of melanoma by inhibiting SIRT7.

Several studies have reported that miR-148b, a member of the miR-148/152 family, has been detected in many types of cancers. This suggests that miR-148b plays an important role in cancer dynamics, including melanoma. In non-small-cell lung cancer, miR-148b suppresses tumor growth and metastasis by regulating the NF-*κ*B and MAPK/JNK pathways [28, 29]. Leong et al. reported that miR-148a/b-3p targets an isoform of RAS-like protein (RALBP1) and thus functions as a tumor suppressor in oral squamous cell carcinomas [30]. A study by Li and colleagues reported that miR-148b expression inhibited the growth and metastasis of endometrial cancer. This is because miR-148b binds the DNMTI gene and this suppresses the tumor [31]. Results from previous studies have reported that expression of miR-148b-3p resulted in potential suppression of renal carcinoma cell growth and invasion by targeting the FGF2-FGFR2 signaling pathway [32]. Moreover, different studies showed that miR-1908, miR-199a-5p, miR-199a-3p, miR-211, miR-204-5p, miR-211-5p, and miR-499a-5p were involved in the process of cell proliferation, invasion, migration, and cell apoptosis in melanoma [33–36]. In summary, these studies revealed that miRNAs play important roles in the development, progression, and tumor-suppressive effects in various types of cancers. However, the specific mechanism of miRNAs in melanoma remains largely unknown. Therefore, the current study was conducted to understand the functions of miRNAs in melanoma.

In this study, it was shown that the expression level of miR-148b was significantly decreased in human melanoma tissues compared to benign nevi. From the results of the cell invasion and proliferation assays, miR-148b was shown to significantly reduce the proliferation, migration, and invasion of cells both *in vitro* and *in vivo*. This data thus indicates that the downregulation of miR-148b is associated with melanoma metastasis and prognosis.

Several studies have linked epigenetic dysregulation to human disease, notably cancer. Findings from several studies have indicated that SIRT7 combines with chromatin and catalyzes the selective deacetylation of H3K18, an epigenetic biomarker of malignant tumors and an indicator of poor prognosis in patients [37, 38]. Through histone deacetylation of H3K18 at specific promoters, SIRT7 controls a tumor-suppressive gene expression program that stabilizes the transformed state of cancer cells. SIRT7 has been shown to promote gastric cancer growth by repressing miR-34a expression through the deacetylation of H3K18 in the promoter region [39, 40]. Additionally, SIRT7 participates in many molecular processes, including rRNA and tRNA synthesis, which enhance ribosome biogenesis that is responsible for tumor cell proliferation. Previous studies have shown that SIRT7 is regulated by various miRNAs in the development and progression of different types of cancer [24, 27]. In the current study, it was established that downregulation of miR-148b, a novel miRNA for regulating SIRT7 in melanoma, contributed to the increased expression of SIRT7. Furthermore, the results showed that miR-148b suppressed the expression of SIRT7 by selectively binding to the 3′UTR of SIRT7. Interestingly, the initial level of miR-148b was greatly declined in melanoma, which might account for the anti-miR-148b inability to raise the level of SIRT7.

Moreover, results from the experiments in this study showed that the inhibition of SIRT7 by miR-148b significantly inhibited the migration, invasion, and proliferation of malignant cells. Contrary to this, the overexpression of SIRT7 reversed the antitumor effect of miR-148b *in vitro*. Therefore, this evidence suggests that the miR-148b/SIRT7 axis has a significant role in the progression of melanoma. However, SIRT7 failed to significantly reverse the antitumor effect of miR-148b in vivo, and this might be attributed to multiple target genes of the miRNA [9], which is involved in a complex interactive biological network. The other target genes which are regulated by miR-148b may participate in the antitumor effect in a mechanism that remains unknown, and further research is needed to better understand this.

## 5. Conclusion

According to the findings of the present study, it was demonstrated that SIRT7 was overexpressed but miR-148b expression level was significantly decreased in melanoma cells and tissues. The expression of SIRT7 was negatively regulated by miR-148b. The proliferation and metastasis of melanoma cells could be inhibited by miR-148b binding to the 3′UTR of SIRT7. However, SIRT7 was shown to reverse the inhibitory effect of miR-148b. These findings indicated that miR-148b plays a protective role in melanoma progression by reducing the expression level of SIRT7. Therefore, miR-148b may be a potential therapeutic target for the treatment of melanoma.

## Figures and Tables

**Table 1:** Sequence of miR-148b and U6.

Name	Nucleotide sequence
miR-148b upstream primer	CAGTGCGTGTCGTGGAG
U6 sense	CTCGCTTCGGCAGCACA
U6 antisense	AACGCTTCACGAATTTGCGT

**Table 2:** Primer sequence of miR-148b, SIRT7, and GAPDH.

Name	Nucleotide sequence
miR-148b forward primer (10 *μ*M)	UCAGUGCAUCACAGAACUUUGU
miR-148b reverse primer (10 *μ*M)	AAAGUUCUGUGAUGCACUGAUU
SIRT7 forward primer (10 *μ*M)	CGTCCGGAACGCCAAATAC
SIRT7 reverse primer (10 *μ*M)	GACGCTGCCGTGCTGATT
GAPDH forward primer (10 *μ*M)	CGGAGTCAACGGATTTGGTCGTATTGG
GAPDH reverse primer (10 *μ*M)	GCTCCTGGAAGATGGTGATGGGATTTCC

**Table 3:** Sequence of wild-type and mutant SIRT7 reporter plasmids.

SIRT7-3′UTR-MT	AGATCCTCATAAAGGCCAAGAAGGGCGGAAAGATCGCCGTGTAATTCTAGATCACGTGCTCGATGAAGAACAGTTGGCACTTTGCAGATGGCCAGTGTCACGGTGAAGGCTGGGTTGCCCCCACGGGTCTAGGGAGAACGAACTCTTTGGGGATGACATTTTCACCGTGACATTTTTA**GCCATTTGTCCTTGAGGAAGCC**CCTGTACAGTCTGCGGTTGTACCCTGATACGGCCTGGCCATCGAGGACACCTGCCCATCCGGCCTCTGTGTCAAGAGGTGGCAGCCGCACCTTTCTGTGAGAACGGAACTCGGGTTATTTCAGCCCCGGCCTGCAGAGTGGAAGCGCCCAGCGGCCTTTCCTCGCTCACCAGGCCAGTCTCAGGGCCTCACCGTATTTCTACTACTACTTAATGAAAAAGTGTGAACTTTATAGAATCCTCTCTGTACTGGATGTGCGGCAGAGGGGTGGCTCCGAGCCTCGGCTCTATGCAGACCTTTTTATTTCTATTAAACGTTTC***GTACAGT***GC***TCTAGAGTCGGGGCGGCCGGCCGCTTCGAGCA***
SIRT7-3′UTR-WT	CCGAAAGGTCTTACCGGAAAACTCGACGCAAGAAAAATCAGAGAGATCCTCATAAAGGCCAAGAAGGGCGGAAAGATCGCCGTGTAAT***TCTAGA***TCACGTGCTCGATGAAGAACAGTTGGCACTTTGCAGATGGCCAGTGTCACGGTGAAGGCTGGGTTGCCCCCACGGGTCTAGGGAGAACGAACTCTTTGGGGATGACATTTTCACCGTGACATTTTTAGCCATTTGTCCTTGAGGAAGCCCCTTGCACTGCTGCGGTTGTACCCTGATACGGCCTGGCCATCGAGGACACCTGCCCATCCGGCCTCTGTGTCAAGAGGTGGCAGCCGCACCTTTCTGTGAGAACGGAACTCGGGTTATTTCAGCCCCGGCCTGCAGAGTGGAAGCGCCCAGCGGCCTTTCCTCGCTCACCAGGCCAGTCTCAGGGCCTCACCGTATTTCTACTACTACTTAATGAAAAAGTGTGAACTTTATAGAATCCTCTCTGTACTGGATGTGCGGCAGAGGGGTGGCTCCGAGCCTCGGCTCTATGCAGACCTTTTTATTTCTATTAAACGTTTCTGCACTGGC***TCTAGA***GTCGGGGCGGCCGGCCGCTTCGAGCAGACATGATAAGATACATTGATGAGTTTGGACAAACCACAACTAGAATGCAGTGAAAAAAATGCTTTATTTGTGAAATTTGTGATGCTATTGCTTTATTTGTAACCATTATAAGCTG

## Data Availability

The data used to support the findings of this study are included within the article.
